# Polydopamine-Based Nanocarriers for Photosensitizer Delivery

**DOI:** 10.3389/fchem.2019.00471

**Published:** 2019-07-12

**Authors:** Yuxuan Xiong, Zushun Xu, Zifu Li

**Affiliations:** ^1^Department of Nanomedicine and Biopharmaceuticals, College of Life Science and Technology, Huazhong University of Science and Technology, Wuhan, China; ^2^Key Laboratory for the Green Preparation and Application of Functional Materials, Hubei Collaborative Innovation Center for Advanced Organic Chemical Materials, Ministry of Education, Hubei University, Wuhan, China; ^3^National Engineering Research Center for Nanomedicine, College of Life Science and Technology, Huazhong University of Science and Technology, Wuhan, China; ^4^Hubei Key Laboratory of Bioinorganic Chemistry and Materia Medica, Huazhong University of Science and Technology, Wuhan, China; ^5^Wuhan Institute of Biotechnology, East Lake High Tech Zone, Wuhan, China

**Keywords:** polydopamine, photosensitizers, drug delivery, photodynamic therapy, nanocarriers

## Abstract

Photodynamic therapy (PDT) has emerged as a non-invasive modality for treating tumors while a photosensitizer (PS) plays an indispensable role in PDT. Nevertheless, free PSs are limited by their low light stability, rapid blood clearance, and poor water solubility. Constructing a nanocarrier delivering PSs is an appealing and potential way to solve these issues. As a melanin-like biopolymer, polydopamine (PDA) is widely utilized in biomedical applications (drug delivery, tissue engineering, and cancer therapy) for its prominent properties, including favorable biocompatibility, easy preparation, and versatile functionality. PDA-based nanocarriers are thus leveraged to overcome the inherent shortcomings of free PSs. In this Mini-Review, we will firstly present an overview on the recent developments of PDA nanocarriers delivering PSs. Then, we introduce three distinctive strategies developed to combine PSs with PDA nanocarriers. The advantages and disadvantages of each strategy will be discussed. Finally, the current challenges and future opportunities of PDA-based PS nanocarriers will also be addressed.

## Introduction

Currently, cancer therapies commonly used in clinical practice, such as chemotherapy (Hu et al., 2016), surgery (Katz et al., 2005), and radiotherapy (Schaue and McBride, 2015), have reached their bottlenecks. Drug tolerance, non-specificity, and unavoidable side effects hinder their further development (Dean et al., 2005; Xiao et al., 2013). As an alternative emerging treatment, photodynamic therapy (PDT) brings a new dawn (Dolmans et al., 2003; Lismont et al., 2017). Compared with existing treatment modalities, PDT possesses numerous incomparable superiorities, including non-/minimal invasiveness, low side effects, and controllability (Rong et al., 2014). The typical process of PDT can be briefly summarized as follows: when the lesion site enriched with sufficient photosensitizers (PSs) is irradiated with light, the PSs are converted from the ground state to the excited (triplet) state. Subsequently, the excited PSs transfer their energy directly to the oxygen and generate toxic reactive oxygen species (ROS), such as singlet oxygen (^1^O_2_) and free radicals, which rapidly kill cancer cells (Calixto et al., 2016). It is not difficult to find that light source, O_2_, and PSs are three indispensable ingredients for PDT (Agostinis et al., 2011). Among them, PSs are a critical element. In recent years, several types of PSs, such as porphyrin- (Jin et al., 2013), chlorin e6 (Ce6)- (Li et al., 2013), and methylene blue (MB)- (Seo et al., 2014) based therapeutic agents, have progressed largely. However, these PSs still suffer from their low light stability, fast body clearance, and poor water solubility (Teng et al., 2013; Shemesh et al., 2014). To address these issues, suitable nanocarriers are necessary for potent PDT.

Polydopamine (PDA), a melanin-like biopolymer, has a strong binding property, which makes it possible to load chemical drugs or dyes bearing aromatic structure through π-π stacking/hydrophobic interaction (Wang X. et al., 2016). Moreover, many active groups (including catechol, amino, and quinone groups) in PDA are able to react with a variety of drug molecules (Liu et al., 2014). Besides, PDA is an excellent coating material that can easily form a multifunctional core–shell nanostructure through facile dip coating of nanoparticles (NPs) in an aqueous solution of dopamine (DA) (Lee et al., 2007). Importantly, bio-inspired PDA possess excellent biocompatibility and biodegradation, which provide a prerequisite for biological application. These charming features make PDA an ideal nanocarrier for PSs.

**Figure 1:**
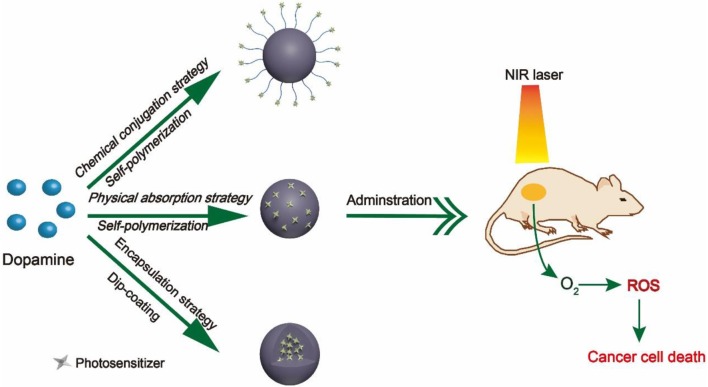
Schematic diagram of three different strategies.

During the past decade, numerous research groups have leveraged PDA as nanocarrier to delivery PSs. According to different interactions between PDA nanocarriers and PSs, we divide these studies into three categories, namely, (1) chemical conjugation strategy, (2) physical absorption strategy, and (3) encapsulation strategy, as summarized in Figure 1. In this Mini-Review, we will highlight the relevant advances regarding PDA-based nanocarriers for PSs and discuss its future prospects and challenges.

## Chemical Conjugation Strategy

**Figure 2:**
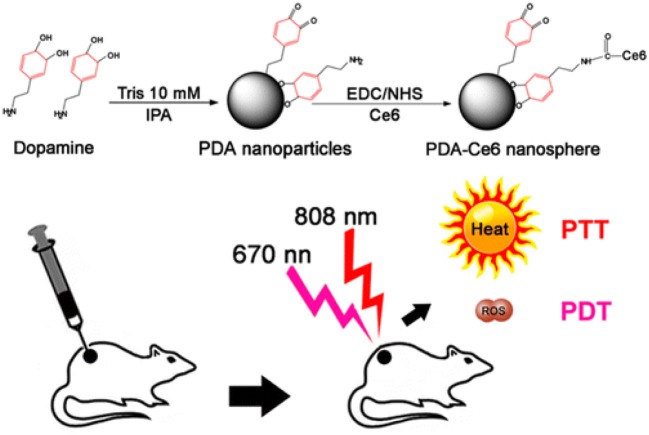
Illustration of the preparation of PDA-Ce6 and their application for PTT and PDT. Reproduced with permission from Zhang et al. (2015). Copyright 2015 American Chemical Society.

Surface functional groups endow PDA NPs with strong chemical reactivity. The covalent conjugation between PDA and amine/thiol-containing molecules is the most widely used reactions in previous studies (Park et al., 2014). Amine-containing molecules react with PDA via Schiff base reaction while thiol-containing molecules react by facile Michel addition reaction (Lee et al., 2009). Owing to the presence of catechol groups, PDA has redox capacity. Hu et al. (2014) firstly modified graphene with PDA. Subsequently, the PDA–graphene reacted with nucleophilic amine groups of folic acid–C_60_ by Michael addition/Schiff base reaction. Due to the high quantum yield of ^1^O_2_ generation of C_60_ and photothermal absorption of graphene, the obtained C_60_-PDA–graphene nanohybrids exhibited synergistic PDT/PTT (photothermal therapy) effect against Hela cancer cells. PDA plays an indispensable role in the whole process, serving as the bridge between graphene and C_60_. Moreover, the introduction of PDA also increased the biocompatibility and hydrophilicity of C_60_-PDA–graphene. Besides, the amino groups on the surface of PDA NPs can also react with PSs containing carboxyl groups (Ce6, porphyrin) by a conventional carbodiimide reaction. Under this premise, Zhang et al. (2015) developed a NP to achieve PDT/PTT combination therapy of tumors (Figure 2). In their work, Ce6 was covalently conjugated onto the surface of PDA. Compared to free Ce6, PDA–Ce6 NPs exhibited higher photo-stability and PDT efficacy. Furthermore, the satisfactory NIR absorption enables PDA to be applied in PTT. Under the irradiation of two different lasers (670 and 808 nm), the PDA–Ce6 NPs demonstrated potent antitumor efficacy both *in vitro* and *in vivo*. To improve the load efficiency of PSs, Yang et al. (2019) selected black phosphorus nanosheets (BP NSs) as the basic nanocarrier, and BP NSs are then coated with PDA and covalently linked with Ce6. Compared to PDA–Ce6, the larger surface area of BP can significantly increase the load of PSs on PDA.

To further potentiate therapeutic efficacy and safety, it is advisable to develop delivery systems with controlled drug release property. Zhan et al. (2019) have skillfully designed and fabricated a thermal-triggered PS release system adopting DNA complementary base pairs. In their work, adenine (A) was firstly modified on the surface of PDA NPs via carbodiimide reaction. Then, thymine (T)-ZnPc spontaneously bound to the surface of A-PDA by simple mixing. Upon 808-nm laser irradiation, the photothermal effect of PDA induced the destruction of A–T bonds, leading to the release of ZnPc. When irradiated with a 665-nm laser, ROS was generated from ZnPc. Based on the characteristics of the tumor microenvironment (TME), the introduction of responsive chemical/biological bonds to construct a TME-responsive (pH-, GSH-, enzyme-responsive) release nanoplatform will greatly improve drug delivery efficiency and reduce side effects.

Chemical conjugation strategy is based primarily on the rich functional groups on PDA; any PS with one of these groups, amine, carboxylic, and thiol, is able to be directly combined with PDA. However, there remain some issues to be addressed. For instance, chemical conjugation may affect the surface properties of PDA, such as hydrophilicity/hydrophobicity, roughness, and surface charge, which will hinder cellular endocytosis. Due to the incomplete reaction and restrained reaction sites, the load efficiency of PSs is relatively low, limiting potent antitumor efficacy of PDT.

## Physical Absorption Strategy

**Figure 3:**
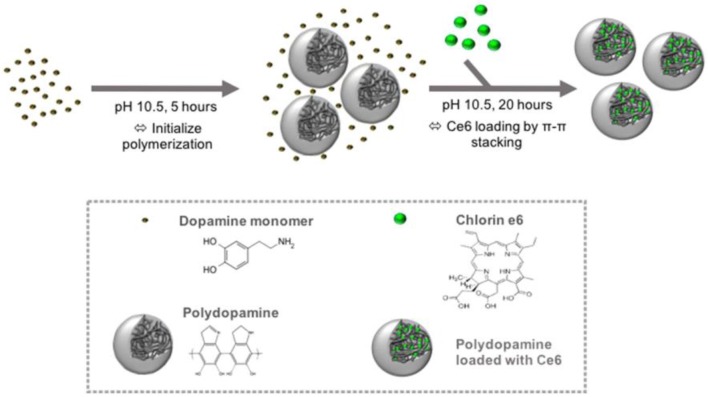
Schematic of the preparation of PDA NPs and loading of hydrophobic PS Ce6 into the polymer matrix of PDA NPs. Reproduced with permission from Poinard et al. (2018). Copyright 2018 American Chemical Society.

The fabrication of PDA NPs involves physical self-assembly, which occurs due to the non-covalent interactions (Dong et al., 2018), including π-π stacking, electrostatic interaction, and hydrogen bonding (Dreyer et al., 2012). Compared with chemical conjugation, it is easier to load PSs onto PDA NPs by non-covalent bonding, since no activation energy is needed in the binding process and the physical absorption is usually carried out in a mild condition. PDA possesses superior capability to load various drugs (Wang X. et al., 2016) or PSs bearing aromatic groups. For instance, Poinard et al. (2018) reported a two-step method to load Ce6 in the polymer matrix of PDA NPs (Figure 3). As expected, the π-π stacking interaction between PDA and Ce6 enabled a prolonged PSs release and enhanced Ce6 stability. Unlike the chemical conjugation, in this system, after Ce6 was loaded onto PDA, the surface properties of PDA had not changed significantly, which was beneficial for cellular uptake. At present, there are many similar studies on loading PSs onto PDA by π-π stacking interactions, such as PDA@IR820 (Wang et al., 2018), PDA@curcumin/Ce6 (Yu et al., 2017), GNR@PDA@MB (Wang S. et al., 2016), etc. Further, to strengthen the specificity of PSs to tumor sites, Han et al. (2016) used PDA NPs as cores and then shielded PDA core with a PS-conjugated hyaluronic acid (HA) coating (PDA@HA-PSs). PDA NPs are thus used as a fluorescence quencher while HA is used as a targeting moiety. In tumor sites, the HA-PS shell was degraded by hyaluronidase (HAase), thereby separating free PSs and enabling the fluorescence recovery and singlet oxygen generation.

The hypoxic environment of tumors represents the biggest obstacle to PDT (Chen et al., 2015). Inspired by the oxygen-carrying properties of red blood cells (RBCs), Zhang et al. (Liu et al., 2018) have skillfully encapsulated hemoglobin (Hb)- and MB (a PS)-loaded PDA NPs into the recombined RBC membranes. Due to the identical origin of RBC membranes, the obtained nanoplatform exhibits outstanding biocompatibility and low immune responses. Importantly, PDA plays a pivotal role, not only as a nanocarrier to load MB but also as an antioxidative factor to protect oxygen-carrying Hb from the oxidation damage during the circulation.

The physical absorption strategy is rather straightforward and does not require any chemical intervention. Moreover, PDA can simultaneously load multiple drugs through non-covalent conjugation to achieve combination therapy. The drug-loaded PDA-based nanocarriers exhibited great drug retaining capability under physiological conditions and could rapidly release the loaded drugs in near-infrared light/low pH environment. However, the reliability of non-covalent bonding and the load efficiency of PSs still need further exploration and improvement.

## Encapsulation Strategy

**Figure 4:**
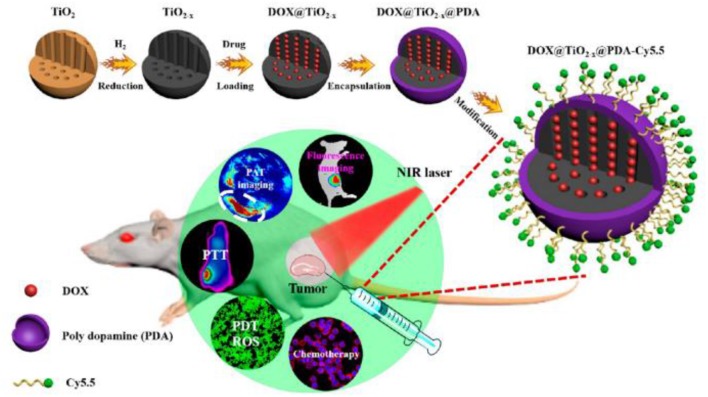
Schematic illustration of the synthetic method and bioimaging-guided triple therapy. Reproduced with permission from Guo et al. (2017). Copyright 2017 American Chemical Society.

Under weak alkaline condition, DA can undergo simple oxidative self-polymerization reaction, and the generated PDA tends to spontaneously deposit on the surface of various materials (Lee et al., 2007). PDA coating is biocompatible and stable *in vivo*, rendering it an ideal platform for biomedical applications. Furthermore, the introduction of PDA shell endows the materials with more functions. Owing to these outstanding features, PDA-based encapsulation strategy has been widely adopted in PDT. For example, Guo et al. (2017) reported a TiO_2−x_ based nanoplatform for dual modal imaging and NIR-triggered chem/photodynamic/photothermal combination therapy (Figure 4). The TiO_2−x_ NPs possessed strong NIR absorption and could induce both hyperthermia and ROS under NIR irradiation. PDA coating behaved as a gatekeeper to avoid premature drug release and achieve a controlled drug release under acidic tumor environment and high temperature.

Indeed, most of the PSs are activated by visible or UV light, which has poor tissue penetration capability, thereby limiting their application in deep-tissue PDT (Wang et al., 2013b). Using NIR-excitable upconversion nanoparticles (UCNPs) as energy donor has been demonstrated to be a valid solution (Wang et al., 2013a). To this end, Cen et al. (2017) designed and prepared a core–shell–shell multifunctional UCNP@SiO_2_-MB@PDA nanoplatform for mRNA detection and PDT/PTT combination therapy. In this nanoplatform, PSs were entrapped in SiO_2_ shell, and the outer PDA shell was constructed through an *in situ* self-polymerization process. Remarkably, the photodynamic performance excited by a 980-nm NIR laser is attributed to the UCNP core. Besides, fluorescently labeled hpDNA was absorbed on PDA shell for sensing tumor-related mRNAs and discriminating cancer cells from normal cells.

To sum up, PDA-based encapsulation strategy is capable of preventing the premature release of PSs in blood circulation effectively, and at the same time, the PDA shell can serve as an active platform for further functionalization, thus assisting cancer PDT. Nevertheless, the PDA shell may hinder the electron transfer of PSs, which will affect the generation of ROS. Furthermore, for organic dye-based PSs, it is usually necessary to introduce a matrix material for assistance, while PDA is only used as a shell. Constructing hollow/capsule PDA NPs to load PSs may be a better method. Besides, the shell thickness of PDA should be tailored well. Due to the broad absorption toward wide range of light, a very thick PDA shell will impede the generation of ROS while a very thin PDA leads to premature leakage of encapsulated PSs.

## Summary and Outlook

PDA-based nanocarriers have a broad application in the field of nanomedicine due to its appealing properties, and the simple construction process gives it great potential for commercial-scale production. In this Mini-Review, we summarized the recent advances of PDA as PS nanocarriers, which are summarized in Table 1. Based on different interactions between PDA and PSs, we divided these systems into three aspects. Considering that PDA is full of functional groups, we first reviewed the studies on PS binding to PDA via chemical bond. Moreover, the PSs rich in aromatic ring easily absorb on the surface of PDA through physical absorption. For PDA-based encapsulation strategy, PDA serves both as a biocompatible coating and a versatile nanoplatform. Different strategies have their own merits and limitations. The chemical conjugation strategy binds PSs to PDA firmly, but the surface properties of PDA will change, which may affect the blood circulation and cellular uptake of NPs. Although the method of physical absorption is quite simple, the loading of PSs strongly depends on the surfaces of PDA. While encapsulation strategy can enhance the stability and functionality of PSs, at the same time, strong π-π stacking will lead to fluorescence quenching and hamper the production of ROS. Generally, different strategies are adopted to construct nanocarriers according to different characteristics of PSs.

**Table 1:** A summary of PDT based on PDA nanostructures.

**The way PDA combines with PSs**	**Structure**	**Size (nm)**	**Laser parameters**	**Therapy**	**Remarks**	**References**
Chemical conjugation	PDA-Ce6	~49	670 nm, 5 min, 50 mW.cm^−2^	PDT/PTT	Enhanced stability and therapeutic effect	Zhang et al., 2015
	rGO@PDA-FA-C_60_	Sheet	Xe lamp, 15 min, 2 W.cm^−2^	PDT/PTT	Excited by a single light	Hu et al., 2014
	Mn_3_O_4_@ PDA-GQD	~100	670 nm, 30 min, 4 mW.cm^−2^	PDT	Dual mode imaging-guided PDT	Nafiujjaman et al., 2015
	PDA-A = T-ZnPc	~88	665 nm, 15 min, LED: 5 W	PDT/PTT	DNA pairing rules, controlled release	Zhan et al., 2019
Physical absorption	Ce6-PDA (load)	~142	665 nm,15 min, 250 mW.cm^−2^	PDT/PTT	Facile two-step method	Poinard et al., 2018
	Ce6@CaCO_3_-PDA-PEG	~168	660 nm,1 h, 5 mW.cm^−2^	PDT	pH sensitivity, biomineralization	Dong et al., 2018
	PDA-PEG@IR820/Fe^3+^	~81	808 nm, 10 min, 1.0 W.cm^−2^	PDT/PTT	Dual imaging and dual therapy	Wang et al., 2018
	GNR@PDA-MB	~80/50	671 nm, 10 min, 30 mW.cm^−2^	PDT/PTT	A promising drug carrier, theranostic	Wang S. et al., 2016
Encapsulation strategy	UCNP@SiO_2_-MB@PDA	~38	980 nm,10 min, 1.0 W.cm^−2^	PDT/PTT	980-nm laser for PDT, mRNA target	Cen et al., 2017
	DOX@TiO_2−X_@PDA-Cy5.5	~637	808 nm, 10 min, 1.0 W.cm^−2^	PDT/Chem/PTT	Triple therapy, NIR/pH-triggered drug release	Guo et al., 2017
	MNPs@hy-PDA-lac	~305	LED 600 nm, 30 min, 8.6 mW.cm^−2^	PDT	Good dispersibility, targeting ability	Shao et al., 2018
	LAP/ICG@PDA-RGD	~59	808 nm, 5 min, 1.2 W.cm^−2^	PDT/PTT	High encapsulation efficiency	Xu et al., 2018

Although significant efforts have been devoted to develop PDA-based nanocarriers for PDT, there are still many crucial issues that need to be addressed. At the mechanism level, the specific structure and polymerization mechanism of PDA is still elusive, limiting rational design and performance. Because of the limited loading sites of PDA, the load efficiency and stability of PSs on PDA need to be enhanced. Moreover, the strong reactivity of PDA is somewhat favorable, but it may have a negative effect in the organism. The long-term toxicity of PDA-based nanocarriers during the circulation and retention in the organism should be further evaluated. With the joint efforts of scientists, we believe that these issues will be addressed in the near future.

## Author Contributions

The manuscript was conceived and prepared by ZL and YX. YX collected papers and contributed to paper writing. ZX and ZL helped to revised the manuscript.

### Conflict of Interest Statement

The authors declare that the research was conducted in the absence of any commercial or financial relationships that could be construed as a potential conflict of interest.
